# Depolarization Ratios of Methane Raman Bands as a Function of Pressure

**DOI:** 10.3390/molecules25081951

**Published:** 2020-04-22

**Authors:** Dmitry Petrov

**Affiliations:** 1 Institute of Monitoring of Climatic and Ecological Systems, Siberian Branch of the Russian Academy of Sciences, 634055 Tomsk, Russia; dpetrov@imces.ru; 2 Tomsk State University, 634050 Tomsk, Russia

**Keywords:** Raman spectroscopy, methane, depolarization ratio

## Abstract

In this work, we measured the intensities of Q-branches of the ν_1_, ν_2_ and ν_3_ bands in the polarized and depolarized methane Raman spectra in the pressure range of 1–60 atm. It was established that the pressure dependence of depolarization ratios of the ν_2_ and ν_3_ bands are negligible. In turn, the depolarization ratio of the ν_1_ band increases with increasing pressure and reaches approximately 0.0045 at 60 atm. These data are more precise than previously published ones because ν_1_ band intensities were determined taking into account the contribution of overlapping lines of ν_3_ band. The presented data will be useful in calculating the methane polarizabilities at high pressure, as well as in calculating methane Raman spectra for measuring the natural gas composition using Raman spectroscopy.

## 1. Introduction

Methane is one of the most abundant molecules on Earth. It is present in atmospheric air, often found in inclusions in minerals, and is also the dominant component of natural gas. Development of Raman gas analysis [1,2,3,4,5,6,7,8,9] brings up the need of information that allows taking into account changes in the methane Raman spectrum under various conditions (temperature, pressure, environment). At present, shifts and broadenings of its fundamental bands [10,11,12,13,14,15], as well as changes in the ratio of peak intensities [13,15,16,17] are known. One of the important characteristics of the Raman bands, along with the position, half-width, and scattering cross-section, is the depolarization ratio (ρ). This value characterizes the symmetry of the vibrations and is defined as the ratio *I*_⊥_/*I_‖_*, where *I*_⊥_ and *I_‖_* are the Raman intensities with orthogonal and parallel orientation of the polarization plane to the polarization plane of the exciting radiation, respectively. As a rule, the higher the symmetry, the lower the degree of depolarization. On the one hand, measuring the depolarization ratio makes it possible to detect intermolecular interactions that lead to distortion of the symmetry of the molecule [18,19]. On the other hand, the exact values of depolarization ratios obtained from the experiment can be used to verify the polarizabilities of the molecules, which, in turn, are the basis for calculating the line intensities.

It is known that in the methane Raman spectrum, the fundamental ν_1_ band (symmetric C–H stretching mode, 2917 cm^−1^) is polarized, i.e., ρ = 0, and ν_2_ (bending mode, 1534 cm^−1^), ν_3_ (asymmetric C–H stretching mode, 3020 cm^−1^), and ν_4_ (bending mode, 1306 cm^−1^) are depolarized bands, i.e., ρ = 0.75 [20]. These data are correct for low pressure, when the molecules do not significantly affect each other. Rose et al. [21] and Wright et al. [22] showed that the depolarization ratio of ν_1_ in pure methane increases with pressure in the range of 1–1000 bar. The main disadvantage of these works is the low signal-to-noise ratio of the experimental setups used, and as a consequence, the high uncertainty of the measured values. The aim of this work is to obtain the depolarization ratios of both the ν_1_ band and the ν_2_ and ν_3_ bands in the pressure range of 1–60 atm using an experimental setup with higher sensitivity.

## 2. Experiment

For research, a Raman spectrometer based on the 90 degree geometry of scattered light collection was used [1]. The gas cell represented a hollow metal cube with a volume of approximately10 cm^3^ having three fused silica windows. A solid-state laser with a wavelength of 532 nm and a power of 1500 mW was used as an excitation source. To collect the scattered light, two f/1.8-lens objectives were used. A holographic notch-filter and a polarizer were installed between them. Rotation of the polarizer allowed us to register radiation with the desired polarization plane. To record Raman spectra, we used an f/1.8-spectrometer equipped with a Hamamatsu CCD array (2048 × 256 pix) with thermoelectric cooling to − 10 °C. Using a diffraction grating of 1600 lines/mm provides simultaneous record of spectrum in the range of 200–3800 cm^−1^ with a dispersion of approximately 1.8 cm^−1^/pixel. Wavenumber calibration was performed using the emission spectrum of neon [23].

Raman spectra of methane were recorded on the equipment described above at pressures from 1 to 60 atm with a step of 5 atm. For each pressure, polarized (*I_‖_*) and depolarized (*I*_⊥_) spectra were recorded. The exposure time at a pressure of 1 atm was 500 s, and for all other pressures it was 100 s. The gas temperature and the air temperature in the laboratory during all experiments were maintained at a level of 298 ± 1 K. The change in gas pressure in the cell during the recording of two spectra with different polarizations was no more than 0.01 atm at a pressure of 1 atm, and no more than 0.1 atm at a pressure of 60 atm. The error of the pressure gauge used was less than 0.1%.

## 3. Results and Discussion

**Figure 1:**
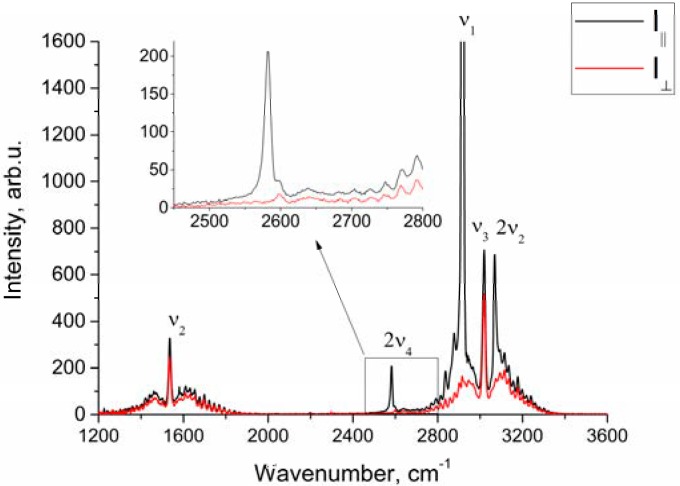
Polarized and depolarized methane Raman spectra at *P* = 50 atm.

**Figure 2:**
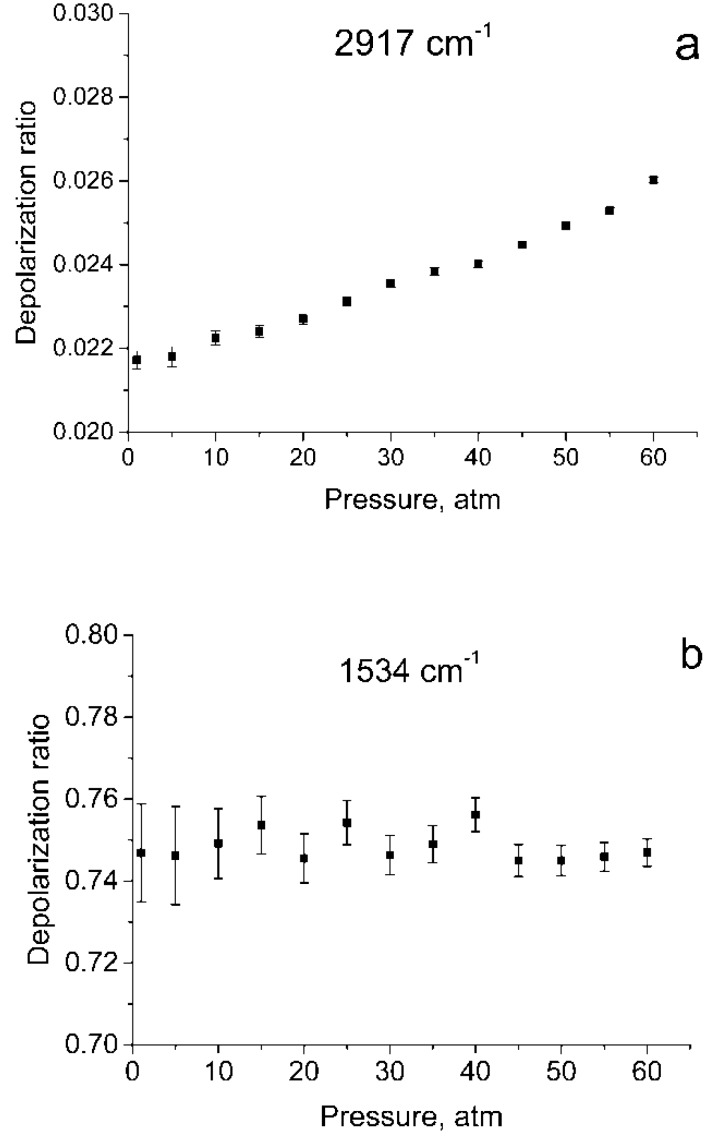

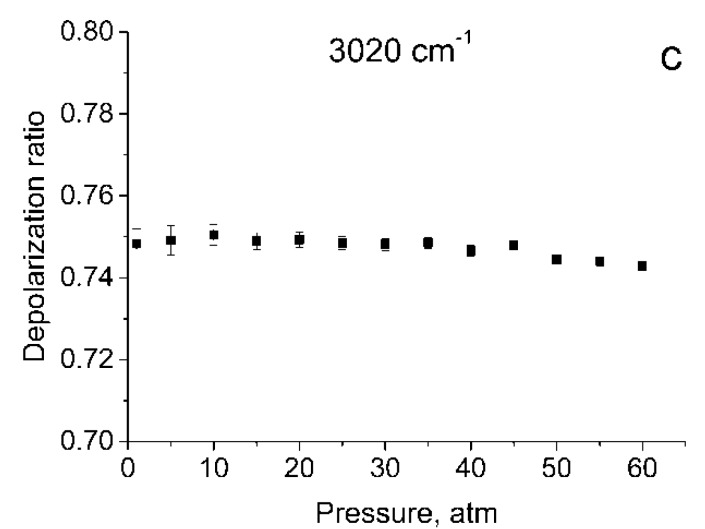
Depolarization ratios for ν_1_ (**a**), ν_2_ (**b**), and ν_3_ (**c**) bands as a function of pressure.

Figure 1 shows the polarized and depolarized methane Raman spectra. It can be seen that only the ν_2_, ν_3_ and, in part, 2ν_4_ bands (F_2_ and E symmetry species [24]) are present in the depolarized spectrum. To obtain the depolarization ratios, the integrated intensities of the Q-branches of ν_1_, ν_2_, and ν_3_ bands in each spectrum were obtained. The intensity of ν_4_ band was too weak to measure. Integration was carried out in the ranges of 2888–2938 cm^−1^, 1515–1555 cm^−1^, and 2990–3040 cm^−1^ for the ν_1_, ν_2_ and ν_3_ bands, respectively. As shown in Figure 2, the pressure dependence of depolarization ratio of the ν_2_ and ν_3_ bands is negligible. In turn, the depolarization ratio of the ν_1_ band increases with pressure.

The error bars shown in Figure 2 were calculated as follows. We believe that in our case, the main source of the error in measuring ρ is the signal fluctuations caused by shot noise and detector noise. To determine these fluctuations, we have additionally recorded five methane Raman spectra at pressures of 1 and 60 atm. From the spectra obtained, standard deviations were estimated of the integral intensities *δ_P_* in the specified spectral ranges for these pressures. For other pressure values, *δ_P_* were estimated by interpolating these data. The errors in measuring the depolarization ratio at each *P* (∆*_P_*) were estimated using Equation (1)

(1) $$\Delta_{P}=\frac{2\delta_{P}}{I_{{∥}_{P}}}$$

where $I_{{∥}_{P}}$ is the integrated intensities in the polarized spectrum obtained at this pressure.

As seen from Figure 1, in the region of 2917 cm^–1^, the ν_1_ band overlaps with the lines of the O-branch of the ν_3_ band. This means that the obtained integrated intensities of ν_1_ band are somewhat different from the true ones. Therefore, the depolarization ratios for ν_1_ ($\rho^{\nu_{1}}$) shown in Figure 2a, have been calculated with an error and should be improved.

**Figure 3:**
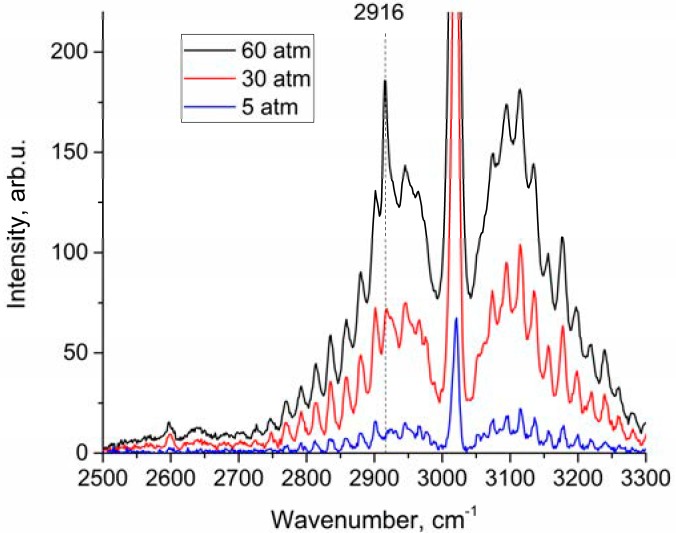
Depolarized methane Raman spectra at 5, 30, and 60 atm.

An analysis of the depolarized spectra showed that the peak in the region of 2916–2917 cm^−1^ increases with pressure, as shown in Figure 3. This peak belongs to ν_1_ band. We estimated changes in the integrated intensities in the range of 2888–2938 cm^−1^ for polarized and depolarized spectra at different pressures. For this purpose, intensities at each pressure (*I_P_*) have been converted into intensities corresponding to the densities of molecules at 1 atm ($I_{P}{}^{\prime }$) using Equation (2):

(2) $$I_{P}{}^{\prime }=\frac{I_{P}}{P/Z(P)}$$

where ***Z(P)*** is the methane compressibility factor at T = 298 K, which is a function of pressure. The virial coefficients for calculating ***Z(P)*** were taken from [25].

**Figure 4:**
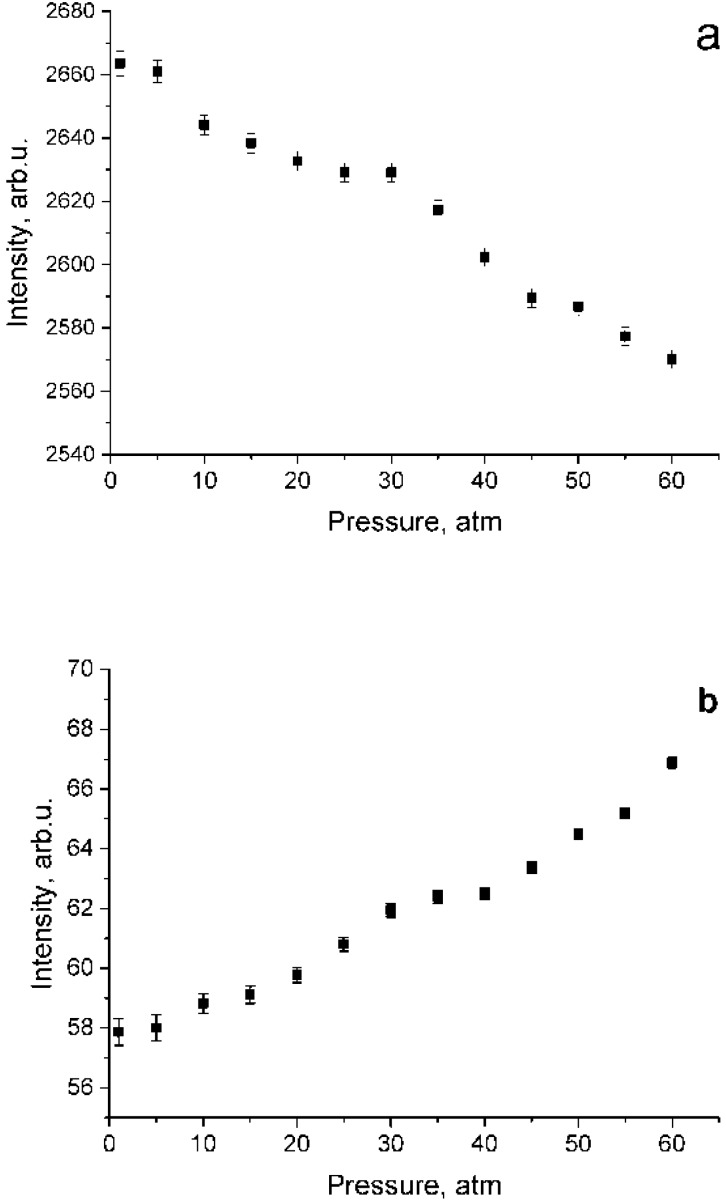
Pressure dependence of integrated intensities in the range of 2890–2940 cm^−1^ for polarized (**a**) and depolarized (**b**) methane spectra which were normalized using Equation (2).

**Figure 5:**
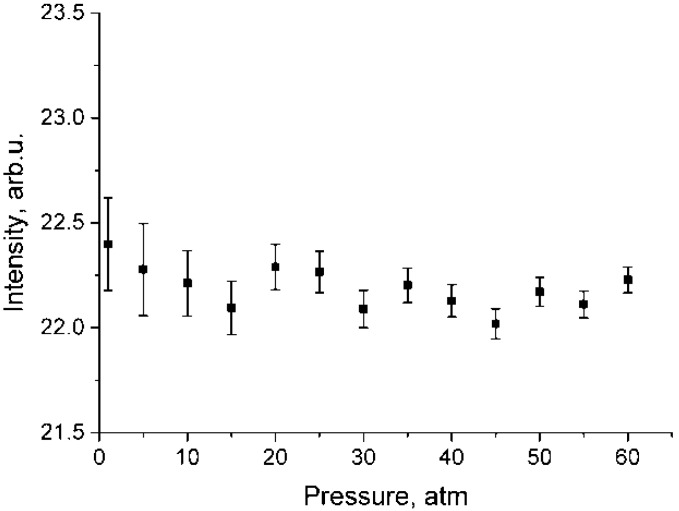
Pressure dependence of integrated intensities of ν_3_ band in the range of 2820–2870 cm^−1^ for depolarized methane spectra which were normalized using Equation (2).

According to the data shown in Figure 4, both a decrease in intensity in the polarized spectrum and an increase in intensity in the depolarized spectrum are observed. This results from lower methane molecule symmetry at high pressure [21,22]. To verify the fact that the increase in the intensity in Figure 4b is not due to the features of the experiment, Figure 5 shows the dependence of the integrated intensity of the depolarized spectrum of the ν_3_ band in the range of 2820–2870 cm^−1^ where there are no ν_1_ lines. Referring to this figure, the intensities after normalization per unit of density is stable. Thus, the data presented in Figure 4b are correct. Note that, as shown in Figure 4, the increase in intensity in the depolarized spectrum is less than its decrease in the polarized one. Taking into account that the intensity ratio of the 2ν_4_/ν_1_ and 2ν_2_/ν_1_ increases with increasing pressure [13], it can be assumed that the decrease in the ν_1_ intensity in the polarized spectrum is caused not only by intensity redistribution to the depolarized spectrum, but also to 2ν_4_ and 2ν_2_ overtones by means of Fermi resonance [24].

The data given in Figure 4b are the sum of intensities of ν_1_ and ν_3_ bands. Thus, they can be represented using Equation (3):

(3) $$I_{⊥}^{\nu_{3}+\nu_{1}}(P)=I_{⊥}^{\nu_{3}}+I_{⊥}^{\nu_{1}}(P)$$

where $I_{⊥}^{\nu_{1}}$ and $I_{⊥}^{\nu_{3}}$ are the intensities for the ν_1_ and ν_3_ bands in the depolarized spectrum, respectively. As seen from Figure 5, pressure dependence of $I_{⊥}^{\nu_{3}}$ is negligible. In addition, according to the calculations [26], at P = 1 atm, $\rho^{\nu_{1}}$ = 0, therefore, $I_{⊥}^{\nu_{3}}(1)$ = 0 and $I_{⊥}^{\nu_{3}+\nu_{1}}(1)=I_{⊥}^{\nu_{3}}$. Having this in mind, we calculated $I_{⊥}^{\nu_{1}}(P)$. The $\rho^{\nu_{1}}$ for various pressures were calculated using Equation (4).

(4) $$\rho^{\nu_{1}}=\frac{I_{⊥}^{\nu_{1}}}{I_{∥}-\frac{4}{3}(I_{⊥}^{}-I_{⊥}^{\nu_{1}})}$$

**Figure 6:**
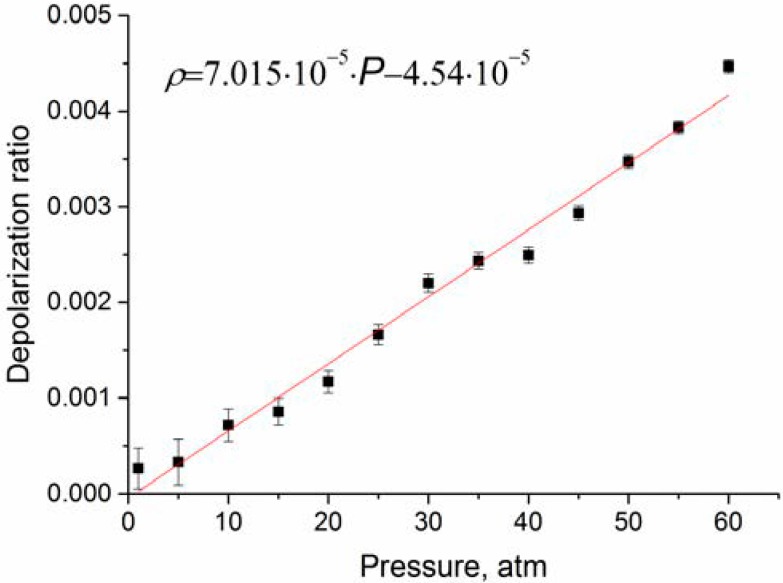
Depolarization ratio of ν_1_ band obtained after correction of the intensities to the contribution of the ν_3_ lines.

As follows from Figure 6, the depolarization ratio increases significantly less compared to the data of Rose et al. [21]. In the studied pressure range, an increase in $\rho^{\nu_{1}}$ is close to a linear dependence. According to [21], $\rho^{\nu_{1}}$ increases with pressure up to approximately 1000 atm. In practice, to analyze the composition of natural gas, it is necessary to take into account changes in spectroscopic characteristics in the pressure range of 1–100 atm. We believe that $\rho^{\nu_{1}}$ at any pressure from this range can be estimated using the function presented in Figure 6.

## 4. Conclusions

The performed studies showed that the dependence of depolarization ratios of the ν_2_ and ν_3_ bands on pressure is negligible. In turn, the degree of depolarization of the ν_1_ band increases with pressure and reaches approximately 0.0045 at 60 atm. These data are significantly lower than those published earlier. This is explained by the fact that in our work we used an experimental setup with higher sensitivity, and also took into account the contribution of the lines of the ν_3_ band located in the region of 2916–2917 cm^−1^. We believe that the obtained data on the pressure dependence of the depolarization ratio, as well as changes in the intensities of the polarized and depolarized spectrum of methane, will improve the accuracy of the calculation of methane polarizabilities. These data will be useful in calculating the Raman spectra of methane at different pressures for analysis of natural gas composition using Raman spectroscopy.
